# The Role of Antiplatelet in the Management of Sickle Cell Disease Patients

**DOI:** 10.7759/cureus.42058

**Published:** 2023-07-18

**Authors:** Sudheeshreddy Naramreddy, Ashish Varma, Amar Taksande, Revat J Meshram

**Affiliations:** 1 Pediatrics, Jawaharlal Nehru Medical College, Datta Meghe Institute of Higher Education and Research, Wardha, IND

**Keywords:** bleeding risk, aspirin, complications, vaso-occlusive events, platelet activation, antiplatelet therapy, sickle cell disease

## Abstract

Sickle cell disease (SCD) is a genetic disorder characterized by abnormal hemoglobin, leading to red blood cell deformities and subsequent vaso-occlusive events. Platelet activation and adhesion play a significant role in the pathophysiology of SCD, contributing to the development of complications such as vaso-occlusive events, stroke, acute chest syndrome, and other manifestations. Antiplatelet therapy has emerged as a potential strategy to mitigate these complications by modulating the platelet function and reducing thrombotic events. This review article provides an overview of antiplatelet therapy's role in managing SCD patients. It discusses the pathophysiological abnormalities in the platelet function in SCD, the rationale for antiplatelet therapy, and the evidence supporting its use in various clinical scenarios. The article explores aspirin as the primary antiplatelet agent in SCD, including its mechanism of action, dosing considerations, and efficacy and safety data. Additionally, it highlights other antiplatelet agents, such as clopidogrel, prasugrel, ticagrelor, and emerging therapies under investigation. Clinical applications of antiplatelet therapy in primary and secondary prevention and the management of acute chest syndrome and other SCD complications are also discussed. Safety considerations are emphasized, including bleeding risk assessment, monitoring, and patient selection for antiplatelet therapy. Finally, the review highlights future research and clinical practice directions, including the development of novel antiplatelet agents, combination therapies, and the integration of antiplatelet therapy with other SCD treatments. Overall, this review provides a comprehensive understanding of the current role of antiplatelet therapy in SCD management, the challenges faced, and future directions for improving patient outcomes.

## Introduction and background

Sickle cell disease (SCD) is a hereditary blood disorder characterized by abnormal hemoglobin, which causes red blood cells to deform into a sickle shape, leading to various clinical manifestations, such as chronic anemia, vaso-occlusive crises, organ damage, and increased susceptibility to infections. This genetic condition predominantly affects individuals of African, Mediterranean, Middle Eastern, and South Asian descent. SCD is a major global health concern affecting individuals and their families [1].

SCD is associated with many complications, ranging from acute vaso-occlusive crises and chronic pain to severe organ damage and life-threatening conditions such as stroke and acute chest syndrome. It is crucial to manage these complications effectively to enhance the quality of life for patients and minimize long-term sequelae. This section will highlight the significance of comprehensive disease management in reducing morbidity and mortality in individuals living with SCD [2,3].

This review article aims to critically evaluate the role of antiplatelet therapy in managing SCD patients. Despite the well-established benefits of antiplatelet agents in other cardiovascular conditions, their potential utility in SCD remains an ongoing research and debate area. This review aims to consolidate existing evidence from clinical studies and laboratory experiments to assess the rationale, efficacy, and safety of antiplatelet therapy in SCD. By comprehensively analyzing the current literature, we hope to offer valuable insights into the potential benefits and limitations of using antiplatelet agents as a therapeutic approach in SCD management.

## Review

Overview of platelet activation and adhesion in SCD

Role of Platelets in SCD Pathogenesis

Traditionally recognized for their role in hemostasis and thrombosis, platelets have been increasingly implicated in SCD pathogenesis. While red blood cells are the primary contributors to vaso-occlusive events in SCD, emerging evidence suggests that platelets also play a significant role in the disease process. Platelets interact with activated endothelial cells and abnormal sickle red blood cells, leading to a cascade of events contributing to vaso-occlusion and tissue damage [4,5].

Abnormalities in Platelet Function in SCD Patients

In SCD patients, platelets exhibit various abnormalities in structure and function. These alterations include increased platelet activation, enhanced adhesion to endothelial cells, altered platelet aggregation, and heightened platelet-monocyte interactions. These abnormalities are influenced by chronic inflammation, oxidative stress, and abnormal hemoglobin. Understanding these aberrations in platelet function is crucial for elucidating the role of platelets in SCD pathophysiology [6,7].

Impact of Platelet Activation and Adhesion on SCD Complications

The activation and adhesion of platelets in SCD contribute to the development of various complications associated with the disease. Platelet activation releases pro-inflammatory and pro-thrombotic factors, such as interleukin-1 (IL-1), exacerbating the inflammatory milieu and promoting vaso-occlusion. Enhanced platelet adhesion to endothelial cells further contributes to the occlusion of blood vessels, leading to tissue ischemia, organ damage, and pain crises. Moreover, platelet-mediated inflammation and oxidative stress play a role in endothelial dysfunction and contribute to the chronic vasculopathy observed in SCD [8].

Understanding the role of platelets in SCD pathogenesis is crucial for identifying potential therapeutic targets. By targeting platelet activation and adhesion, it may be possible to mitigate the complications associated with SCD and improve patient outcomes. Antiplatelet therapy represents a potential strategy for modulating platelet function and reducing vaso-occlusive events in SCD patients. The following sections will explore the rationale for utilizing antiplatelet therapy in SCD, the various antiplatelet agents used, their clinical applications, and safety considerations [9].

The rationale for antiplatelet therapy in SCD

Potential Benefits of Antiplatelet Therapy

Antiplatelet therapy holds promise as a potential therapeutic approach to managing SCD. By targeting platelet activation and aggregation, antiplatelet agents have the potential to mitigate vaso-occlusive events, reduce inflammation, and improve microvascular blood flow. The potential benefits of antiplatelet therapy in SCD include the following:

Prevention of vaso-occlusive events: Antiplatelet agents are crucial in preventing vaso-occlusive events, a hallmark of SCD. These events occur when sickled red blood cells obstruct blood vessels, leading to tissue ischemia and pain crises. Antiplatelet therapy inhibits platelet activation and adhesion, thereby reducing the formation of thrombi and preventing the occlusion of blood vessels. By maintaining blood flow and preventing vaso-occlusive events, antiplatelet therapy can alleviate pain, improve tissue perfusion, and enhance overall patient outcomes in SCD [10].

Modulation of inflammatory processes: In addition to their role in thrombosis, activated platelets also contribute to the inflammatory response in SCD. Platelets release pro-inflammatory cytokines and chemokines, promoting the recruitment and activation of immune cells and exacerbating the inflammatory cascade. Antiplatelet therapy has the potential to attenuate inflammation by inhibiting platelet activation and the subsequent release of inflammatory mediators. By modulating the inflammatory processes, antiplatelet agents may help reduce the severity of inflammatory complications in SCD, such as acute chest syndrome and organ damage [11].

Improvement of microvascular blood flow: The adhesion and aggregation of activated platelets contribute to the occlusion of microvasculature in SCD, leading to tissue ischemia and impaired organ function. Antiplatelet agents can improve microvascular blood flow by inhibiting platelet adhesion and aggregation. This improved blood flow enhances tissue oxygenation, reduces tissue damage, and promotes better organ function. By optimizing microvascular blood flow, antiplatelet therapy may positively impact various SCD-related complications, including leg ulcers, priapism, and other manifestations associated with impaired perfusion [12].

Mechanisms of Action of Antiplatelet Agents

Antiplatelet agents exert their effects through various mechanisms, targeting different aspects of platelet function to reduce platelet activation, aggregation, and adhesion. In SCD, several commonly used antiplatelet agents, including aspirin, clopidogrel, prasugrel, and ticagrelor, have demonstrated efficacy in managing platelet-related complications [13]. Aspirin, a well-known antiplatelet agent, exerts its action by irreversibly inhibiting the cyclooxygenase-1 (COX-1) enzyme. By doing so, aspirin reduces the production of thromboxane A2, a potent platelet activator. Thromboxane A2 promotes platelet aggregation and vasoconstriction, and by inhibiting its synthesis, aspirin effectively decreases platelet aggregation and the subsequent formation of blood clots [14].

Clopidogrel, prasugrel, and ticagrelor belong to a class of drugs known as P2Y12 receptor inhibitors. These agents specifically target the P2Y12 receptor on platelets, crucial in amplifying platelet activation and aggregation. By inhibiting the P2Y12 receptor, these drugs effectively reduce platelet reactivity and aggregation, thereby decreasing the risk of thrombotic events [15]. Clopidogrel is a prodrug that requires activation by liver enzymes to exert its antiplatelet effects. Once activated, it irreversibly binds to the P2Y12 receptor, inhibiting platelet activation and aggregation [16]. Prasugrel, similar to clopidogrel, is also a prodrug that requires hepatic activation. It undergoes conversion to its active metabolite, which has a more potent and rapid onset of action in inhibiting platelet function through P2Y12 receptor blockade [17]. Conversely, ticagrelor directly and reversibly binds to the P2Y12 receptor, inhibiting platelet activation and aggregation. It has a rapid onset of action and does not require hepatic activation such as clopidogrel and prasugrel [18].

These antiplatelet agents, in various combinations or as monotherapy, have demonstrated efficacy in reducing platelet reactivity and preventing thrombotic events in SCD patients. However, the antiplatelet agent and its optimal use should be tailored to the individual patient, considering factors such as bleeding risk, drug interactions, and potential side effects. Close monitoring and personalized approaches are essential to ensure the safety and efficacy of antiplatelet therapy in SCD management [19].

Evidence Supporting the Use of Antiplatelet Therapy in SCD

Primary prevention of vaso-occlusive events: Aspirin has been studied for its role in primary prevention in children with SCD. Some studies have shown a reduced incidence of vaso-occlusive events and acute chest syndrome with low-dose aspirin therapy [20].

Secondary prevention of stroke: Regular blood transfusion therapy is the standard of care for children with SCD who have had an ischemic stroke. However, adjunctive antiplatelet therapy, such as aspirin, may be considered in specific cases [21].

Management of acute chest syndrome: Antiplatelet agents, such as aspirin and ticlopidine, have shown potential benefits in reducing the incidence and severity of acute chest syndrome episodes in SCD patients [22]. While the available evidence suggests potential benefits, it is important to note that antiplatelet therapy in SCD is not without risks. Bleeding complications and individual patient factors must be considered when determining the appropriateness and dosing of antiplatelet therapy in SCD. Further research, including large-scale clinical trials, is needed to establish antiplatelet agents' optimal use, safety, and efficacy in managing SCD.

Antiplatelet agents used in SCD

Aspirin

Mechanism of action: Aspirin is a widely used antiplatelet agent with a well-established mechanism of action. It irreversibly inhibits the COX-1 enzyme, synthesizing thromboxane A2, a potent platelet activator. By inhibiting COX-1, aspirin decreases the production of thromboxane A2, thereby reducing platelet aggregation and vasoconstriction [23].

Dosing considerations: The recommended aspirin dose for children with SCD to prevent vaso-occlusive events is typically 2-5 mg/kg/day. The initiation and duration of aspirin therapy should be determined in consultation with a healthcare provider. When determining the appropriate dose, it is essential to consider factors such as the child's age, weight, and individual risk profile [24].

In SCD patients who have experienced an ischemic stroke, aspirin therapy is often used as adjunctive therapy in combination with regular blood transfusions. The dosing of aspirin in this context is typically 1-5 mg/kg/day. However, the specific regimen should be individualized based on the patient's condition, clinical factors, and the treatment team's guidance [25]. It is important to note that dosing considerations may vary among different guidelines and individual patient circumstances. Factors such as the severity of SCD, concurrent treatments, and other medical conditions may influence the dosing decisions. Therefore, healthcare professionals must provide personalized guidance based on the latest evidence and clinical expertise.

Efficacy and Safety Data in SCD Patients

Clinical trials and observational studies have demonstrated the potential benefits of low-dose aspirin therapy in children with SCD for primary prevention. These studies suggest that aspirin may reduce the incidence of vaso-occlusive events and acute chest syndrome. However, the precise magnitude of the benefit and the optimal duration of therapy require further investigation. Ongoing research aims to clarify the long-term effects and establish evidence-based guidelines for the use of aspirin to prevent complications in SCD [20,26-29].

Aspirin has also been investigated as a preventive measure for acute chest syndrome, a severe complication of SCD characterized by pulmonary inflammation and vaso-occlusion. Preliminary studies have shown that aspirin therapy may decrease the incidence and severity of acute chest syndrome episodes. However, additional research is needed to validate these findings and determine the precise role of aspirin in preventing and managing acute chest syndrome in SCD patients [30].

Regarding safety, aspirin is generally well-tolerated in most individuals with SCD. However, it is important to consider the potential risks, particularly the increased bleeding tendency associated with aspirin use. Close monitoring for signs of bleeding or adverse effects is crucial, especially in patients with a history of bleeding disorders or those at high risk of bleeding complications. The decision to initiate aspirin therapy should be individualized, considering the patient's clinical characteristics, risk factors, and consultation with healthcare professionals experienced in SCD management [31]. It is important to acknowledge that the use of aspirin in the management of SCD should be approached with caution and tailored to each patient's specific needs. Further research, including long-term studies and randomized controlled trials, is warranted to establish aspirin's optimal dosing, efficacy, and safety profile in managing SCD. This research will provide more robust evidence and facilitate the development of evidence-based guidelines for using aspirin and other antiplatelet therapies in the comprehensive care of individuals with SCD.

Other antiplatelet agents

Clopidogrel

Clopidogrel is an antiplatelet agent that acts as a P2Y12 receptor inhibitor, thereby blocking the activation of platelets by adenosine diphosphate (ADP). The P2Y12 receptor is crucial for platelet aggregation and activation. By inhibiting this receptor, clopidogrel reduces platelet aggregation and activation, decreasing the risk of thrombotic events [32].

In SCD, clopidogrel has been investigated as an adjunctive therapy, particularly in patients with a history of vaso-occlusive events or stroke. The rationale behind its use is to inhibit platelet activation and aggregation further, potentially reducing the incidence and severity of these complications in SCD. However, it is important to note that evidence supporting the use of clopidogrel in SCD is limited, and further research is required to establish its efficacy and safety profile, specifically in this patient population [33].

To establish the efficacy and safety of clopidogrel in SCD, larger-scale clinical trials with well-designed protocols and longer follow-up periods are warranted. These studies should focus on evaluating the impact of clopidogrel on vaso-occlusive events, stroke prevention, and other relevant outcomes in SCD patients. Factors such as dosing considerations, potential drug interactions, and the impact on bleeding risk must be thoroughly assessed [34].

Prasugrel

Prasugrel is a P2Y12 receptor inhibitor that functions by blocking platelet activation and aggregation. It exerts its effect by inhibiting the activation of platelets and preventing the formation of blood clots. In SCD, prasugrel has been studied for its potential clinical application in managing vaso-occlusive events, a hallmark of SCD complications. However, it is important to note that evidence supporting the use of prasugrel, specifically in SCD, is limited [35].

Although prasugrel has been evaluated in other cardiovascular conditions, its efficacy, safety, and optimal dosing in SCD patients have not been extensively studied. Limited evidence suggests that prasugrel may have a role in reducing platelet activation and subsequent vaso-occlusive events in SCD. However, more research is required to determine its effectiveness and safety profile in this patient population [36]. Future studies should focus on evaluating the efficacy of prasugrel in reducing vaso-occlusive events in SCD, as well as its potential benefits compared to other antiplatelet agents. Additionally, investigations into the optimal dosing regimen and potential interactions with other medications commonly used in SCD management are warranted.

Ticagrelor

Ticagrelor is a P2Y12 receptor inhibitor that exerts its antiplatelet effects by blocking the P2Y12 receptor, thereby inhibiting platelet activation and aggregation. By preventing the binding of adenosine diphosphate (ADP) to its receptor on platelets, ticagrelor interferes with the signaling pathways involved in platelet activation, ultimately reducing platelet-mediated thrombosis [37].

In the context of SCD, ticagrelor has shown promise in preclinical studies and small clinical trials. These studies have demonstrated its potential benefits in reducing vaso-occlusive events and improving microvascular blood flow in SCD patients. The inhibition of platelet activation and aggregation by ticagrelor may help prevent the formation of thrombi, alleviate vaso-occlusive episodes, and improve tissue perfusion in affected individuals [38]. However, it is important to note that the use of ticagrelor in SCD is still investigational, and further research is required to establish its efficacy, safety, and optimal dosing in this patient population. Large-scale clinical trials are needed to evaluate its effectiveness in reducing specific SCD complications and to determine the potential benefits and risks associated with its use. The potential interactions with other medications commonly used in SCD management should also be considered.

Other Agents Under Investigation

Apart from the aforementioned agents, there are ongoing investigations into the potential use of other antiplatelet agents, such as prasugrel, cangrelor, and vorapaxar, in SCD. These agents target different pathways involved in platelet activation and aggregation and may offer novel treatment options. However, their specific role in SCD management is still being explored, and more research is needed to determine their efficacy, safety, and optimal use [39].

It is important to note that these alternative antiplatelet agents in SCD should be cautiously approached and tailored to individual patient characteristics. Close monitoring for bleeding complications and consultation with healthcare professionals experienced in SCD management is crucial. Further research and well-designed clinical trials are necessary to establish these agents' efficacy, safety, and optimal use in managing SCD [40].

Clinical applications of antiplatelet therapy in SCD

Primary Prevention of Vaso-Occlusive Events

Vaso-occlusive events, characterized by the obstruction of small blood vessels by sickled red blood cells, are a major cause of morbidity in SCD. Antiplatelet therapy, particularly with low-dose aspirin, has been investigated to prevent SCD. Studies have suggested that low-dose aspirin may reduce the incidence of vaso-occlusive events and acute chest syndrome in children with SCD. However, further research is needed to determine aspirin therapy's optimal dosing, duration, and long-term effects in this context [10].

Secondary Prevention of Stroke

Stroke is a serious complication of SCD, particularly in children. Regular blood transfusion therapy is the standard of care for secondary prevention in SCD patients with a history of ischemic stroke. However, adjunctive antiplatelet therapy, such as aspirin, may be considered in certain cases. The use of aspirin in secondary prevention of stroke should be based on individual patient characteristics, risk factors, and consultation with a healthcare professional [41].

Management of Acute Chest Syndrome

Acute chest syndrome is a potentially life-threatening complication of SCD characterized by chest pain, fever, and pulmonary infiltrates. Antiplatelet agents, such as aspirin and ticlopidine, have shown potential benefits in reducing the incidence and severity of acute chest syndrome episodes. These agents may help prevent platelet activation and aggregation within the pulmonary vasculature, potentially reducing inflammation and vaso-occlusion. However, further research is needed to establish their efficacy, optimal dosing, and safety profile in this context [42].

Use in Other Complications of SCD

Antiplatelet therapy has also been explored for its potential benefits in managing other complications associated with SCD, such as priapism (prolonged painful erection) and leg ulcers. Although the evidence is limited, antiplatelet agents, including aspirin, have shown some promise in reducing the frequency and severity of priapism episodes and promoting wound healing in leg ulcers. However, additional research is needed to determine antiplatelet therapy's optimal use and long-term effects in these specific complications [43].

It is important to note that antiplatelet therapy in SCD should be individualized based on patient characteristics, clinical factors, and consultation with healthcare professionals experienced in SCD management. Antiplatelet therapy should be considered within the broader context of comprehensive SCD care, including supportive therapies, hydroxyurea, and regular medical follow-up. Further research, including large-scale clinical trials, is necessary to establish antiplatelet agents' optimal use, safety, and efficacy in managing SCD and its associated complications [44].

Safety and considerations in antiplatelet therapy

Bleeding Risk in SCD Patients

SCD is associated with an increased risk of bleeding due to various factors, such as platelet dysfunction, vessel fragility, and the potential presence of other comorbidities. It is crucial to assess the bleeding risk in each patient before initiating antiplatelet therapy. Factors that may increase bleeding risk include a history of bleeding disorders, active bleeding, recent surgery, or concomitant use of other medications that affect bleeding, such as nonsteroidal anti-inflammatory drugs (NSAIDs). Hemostatic balance and individual patient factors should be carefully considered [45].

Monitoring and Management of Bleeding Complications

Close monitoring for bleeding complications is essential when using antiplatelet therapy in SCD. Patients should be educated about the signs and symptoms of bleeding, including excessive bruising, prolonged bleeding from minor cuts, blood in stools or urine, and gum bleeding. Regular follow-up visits and laboratory monitoring may be necessary to assess platelet function, coagulation parameters, and hemoglobin levels. If bleeding complications occur, prompt evaluation and appropriate management, such as platelet transfusion or local measures, should be initiated [46].

Patient Selection and Personalized Approach to Antiplatelet Therapy

The selection of patients for antiplatelet therapy in SCD should be based on individual assessment, considering the specific clinical scenario, patient characteristics, and bleeding risk. A personalized approach is necessary to balance the potential benefits of antiplatelet therapy with the risk of bleeding complications. This includes considering factors such as age, disease severity, concurrent medications, comorbidities, and other risk factors for bleeding. Shared decision-making between healthcare professionals and patients is crucial in determining the appropriate and individualized antiplatelet therapy approach [47].

Additionally, it is important to consider potential drug interactions between antiplatelet agents and other medications commonly used in SCD management, such as hydroxyurea or anticoagulants. Close collaboration between healthcare providers caring for SCD patients is necessary to ensure comprehensive management and minimize potential drug interactions [48]. Furthermore, it is worth noting that evidence supporting the use of antiplatelet therapy in SCD is still evolving. Ongoing research and well-designed clinical trials are needed to elucidate further the optimal use, safety, and efficacy of antiplatelet agents in managing SCD.

Future directions and emerging therapies

Novel Antiplatelet Agents in Development

Researchers are actively exploring the development of novel antiplatelet agents specifically targeted for SCD. These agents aim to address the unique pathophysiology and platelet abnormalities associated with SCD. Various targets and mechanisms of action are being investigated, such as targeting specific adhesion molecules or inhibiting platelet activation pathways. Preclinical studies and early-phase clinical trials are underway to evaluate these novel agents' safety, efficacy, and optimal dosing [49].

Combination Therapies and Potential Synergistic Effects

Combination therapies involving antiplatelet agents and other drugs or interventions are being explored to maximize the therapeutic benefits of SCD management. For example, combination therapy with antiplatelet agents and hydroxyurea, the standard of care for SCD, may offer synergistic effects in reducing vaso-occlusive events and improving overall outcomes. Additionally, combination therapy with antiplatelet agents and other disease-modifying agents, such as gene therapy or targeted therapies, is being investigated for their potential additive or synergistic effects [50].

Role of Antiplatelet Therapy in Conjunction with Other SCD Treatments

Integrating antiplatelet therapy into comprehensive SCD management is an area of ongoing research. Future studies will focus on defining the optimal role of antiplatelet agents in conjunction with other treatments, such as regular blood transfusions, novel disease-modifying therapies, and supportive care measures. Understanding the potential interactions, additive effects, and long-term outcomes of combining antiplatelet therapy with these interventions is crucial for developing evidence-based treatment strategies [51]. Furthermore, large-scale clinical trials and long-term studies are needed to further investigate antiplatelet therapy's safety, efficacy, and optimal use in various clinical scenarios, including primary prevention, secondary prevention, and management of specific complications. These studies should involve diverse patient populations and consider genetic variations, disease phenotypes, and coexisting medical conditions.

## Conclusions

In conclusion, antiplatelet therapy's role in managing SCD holds promise in mitigating the complications associated with platelet activation and aggregation. Current evidence suggests that antiplatelet agents, particularly aspirin, can reduce the incidence and severity of vaso-occlusive events, stroke, and acute chest syndrome in SCD patients. However, the field is still evolving, and further research is needed to determine optimal dosing, long-term effects, and safety profiles of antiplatelet therapy in SCD. Close monitoring for bleeding complications and personalized patient selection are crucial considerations. Future directions in research and clinical practice include the development of novel antiplatelet agents, exploring combination therapies, and integrating antiplatelet therapy with other SCD treatments. Continued collaboration and large-scale clinical trials will contribute to advancing the field and improving outcomes for individuals living with SCD.
